# A randomised, patient-assessor blinded, sham-controlled trial of external non-invasive peripheral nerve stimulation for chronic neuropathic pain following peripheral nerve injury (EN-PENS trial): study protocol for a randomised controlled trial

**DOI:** 10.1186/s13063-016-1709-2

**Published:** 2016-12-06

**Authors:** Selina Johnson, Andreas Goebel, Roberta Richey, Emily Holmes, Dyfrig Hughes

**Affiliations:** 1 The Walton Centre NHS Trust, Liverpool, UK; 2 The University of Liverpool, Liverpool, L69 3BX UK; 3 Centre for Health Economics and Medicines Evaluation, Bangor University, Bangor, Gwynedd LL57 2PZ UK

**Keywords:** Peripheral nerve injury, Neuropathic pain, External non-invasive peripheral nerve stimulation, Chronic pain

## Abstract

**Background:**

Eight percent of people in the UK are estimated to have persistent (chronic) neuropathic pain, and for many there is no effective treatment. Medications are the most common first-line treatment but often have limited benefit or adverse events. Surgical treatments, such as spinal cord stimulation, are then often considered. External non-invasive peripheral nerve stimulation (EN-PENS) is a form of electrical stimulation that involves placing a pen-shaped electrode onto the skin, which can be easily self-administered by patients. Observational studies suggest that EN-PENS may relieve pain for people with localised neuropathic pain; however, there is currently no evidence from controlled trials to confirm the efficacy and confidently determine the effect size for patients with longstanding neuropathic pain.

**Methods:**

EN-PENS is a single-site, blinded, randomised controlled parallel-group superiority add-on trial with a 1:1 allocation ratio, designed to evaluate the efficacy of treatment versus control treatment in 76 patients with longstanding neuropathic pain following peripheral nerve injury. Patients with moderate to -severe neuropathic pain following peripheral nerve injury will be randomised to receive either the active or control treatment, followed by an optional treatment extension or treatment switch to the alternative treatment arm. The primary outcome is average 24-h pain intensity recorded on an 11-point (0–10) numerical rating scale, averaged over the last 7 days of treatment.

**Discussion:**

Study results will be used to inform potential treatment efficacy and cost-effectiveness of EN-PENS for this population group.

**Trial registration:**

ISRCTN53432663. Registered on 7 July 2016.

## Background

Neuropathic pain can arise peripherally or centrally as a direct consequence of a lesion or disease affecting the somatosensory system [1]. Pain can persist long after the initial cause has resolved. An estimated 8 in every 100 people in the UK have persistent chronic neuropathic pain [2, 3]. Neuropathic pain is often severely debilitating, it impinges on the physical, economic and emotional well-being of patients and is associated with low quality of life (QOL) [4].

Neuropathic pain is very challenging to manage because of the heterogeneity of its aetiology and underlying mechanisms [5].

Current management guidelines are heavily weighted on pharmacotherapy, often with modest outcomes [6, 7]. When pharmacotherapy management is measured as sub-optimal, either due to adverse events or insufficient pain relief, next-line therapy options include surgical lesioning or neuromodulation therapy [8]. A disadvantage of surgical lesioning is that it is non-adjustable and not reversible. Many procedures such as neurotomies have been discontinued in clinical practice. Neuromodulation therapy involves using electrical or chemical technology that acts directly upon nerves to alter or modulate nerve activity. The majority of neuromodulation technologies are invasive. Treatments such as spinal cord stimulation (SCS), dorsal root ganglion (DRG) stimulation and deep brain stimulation (DBS) require surgical implants. Less invasive technologies such as percutaneous electrical nerve stimulation (PENS) involve electrical stimulation of needles inserted within the skin to target peripheral nerves [9]. A disadvantage of such therapies is that the patient will require invasive procedures to obtain benefits [10, 11].

Non-invasive neuromodulation technologies include external non-invasive peripheral nerve stimulation (EN-PENS) and transcutaneous electrical nerve stimulation (TENS). EN-PENS is a neuromodulation technology in which an electrode is positioned on the skin over the injured nerve, and low-frequency electrical stimulation (1–2 Hz) is applied to the nerve. This stimulation mode aims to achieve long-lasting analgaesia through a specific mechanism, preferential activation of superficial nociceptive A-delta fibres inducing long-term depression (LTD) of synaptic strength [12–15]. These effects can last up to a few days [16], rendering this an attractive stimulation mode for intermittent applications. Patients can also easily be taught to self-administer treatment safely at home.

In contrast to EN-PENS, TENS, another non-invasive form of neuromodulation, typically activates A-beta fibres not involved in LTD when used in conventional mode (50–100 Hz typically) [17]. Although when used at very low frequency it may theoretically elicit LTD, it then requires much higher current to overcome the low current density under the electrode [12, 13, 15, 18]. Published evidence regarding the effects of LTD is largely supported by animal and laboratory-based studies, but as yet clinical human study evidence is lacking.

The first prospective cohort study on the use of EN-PENS in neuropathic pain demonstrated significant pain reduction in persons with chronic neuropathic pain post peripheral nerve injury (PPNI) or complex regional pain syndrome (CRPS) (*n* = 20, prospectively assessed, 2.8 numerical rating scale (NRS) points average reduction, CI 1.6–4.0, *p* < 0.001). No treatment-related adverse events were reported. Significant improvements in QOL (measured using the EuroQol (EQ)-5D-5 L) and function (using the Brief Pain Inventory Interference Subscale and the task-specific scale) were also reported [19]. This study identified a trend toward patients with PPNI demonstrating greater reductions in pain for longer periods than patients with CRPS.

Further review of long-term EN-PENS efficacy (*n* = 5, average follow-up 3.5 years) indicated treatment benefits beyond those immediately following treatment [20]. The analgaesic effect remained stable for at least 1 year, with one to two treatments/day, with no evidence of tolerance.

This initial study provides proof of concept for the efficacy of EN-PENS in reducing pain in persons with PPNI and CRPS who have moderate to severe pain intensity, with no adverse events reported. These data suggest that this treatment may improve function and QOL. Therefore, the potential of EN-PENS as a safe, effective and cost-effective treatment option for those with chronic neuropathic pain, for whom effective treatment options may be limited, warrants further consideration.

Aside from the initial study described, current evidence for this modality exists only in the form of conference abstracts describing case series [21–25]. Results report positive responses in terms of immediate pain relief following treatment primarily in patients with neuropathic pain.

It is important to consider this potentially effective treatment in a randomised controlled trial (RCT) with a larger group of patients. Previous study results do not allow for the exclusion of possible moderate long-lasting changes in the pain intensity after stopping EN-PENS treatment [19]; therefore, a crossover RCT design is not feasible.

The primary objective of this trial is to establish clinical efficacy and a more confident estimate of the effect size of EN-PENS treatment to reduce pain in patients with moderate to severe neuropathic pain associated with definite or probable peripheral nerve injury.

Secondary objectives are to evaluate the impact of this treatment in terms of QOL and day-to-day function. Further objectives are to gain a better understanding of this modality in respect to mood, self-efficacy (confidence to perform abilities in the presence of pain), reduction of allodynia, potential mode of action, cost-effectiveness and health care resource use (e.g. whether treatment can negate the need for more complex surgical treatments and/or reduce the need for drug treatment).

## Methods

### Trial design

The EN-PENS trial is a single-site, blinded, randomised controlled parallel-group superiority add-on trial with a 1:1 allocation ratio.

**Fig. 1:**
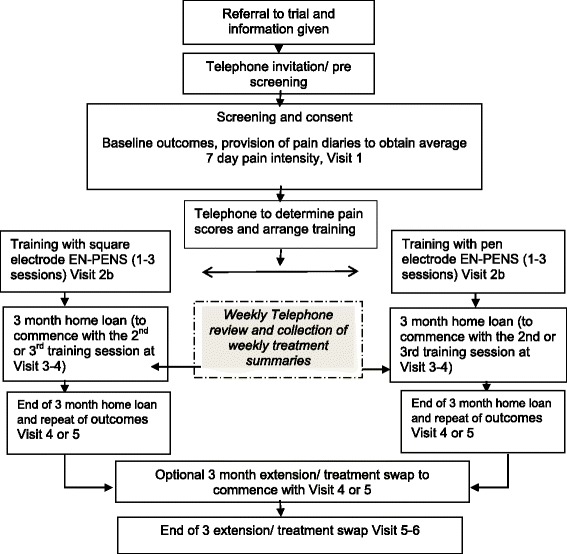
EN-PENS Study flow chart

Following screening, 76 patients will be randomised to receive either active or control (sham) treatment (38 in each treatment arm). Participants and the research nurses providing the training and undertaking the study assessments will be blinded. Participants will receive training and supportive materials on use of the neuromodulation stimulator (active or control). Once competent at using the EN-PENS machine, participants will enter the 3-month home loan treatment phase. At the end of the treatment phase, a 3-month optional choice of treatment extension or swap to the other treatment arm will be offered (see the flow diagram of Fig. 1). The study duration will be 36 months (from study setup to analysis).

### Setting

This will be a single-site study conducted at a neurosciences research centre in a tertiary specialist neurosciences hospital providing a pain management service.

### Recruitment and screening

Patients will be identified through pain clinics at the study site for the trial. Nearby secondary care pain clinics and orthopaedic departments have been made aware of the study and, as with current practice, will be able to refer patients to the centre for specialised treatment not currently offered within secondary care. Potentially eligible patients will be given an information leaflet regarding the study. Patients will contact or be contacted 1 week after receiving the leaflet to ascertain if they have any questions and arrange a screening appointment. Screening in respect to inclusion and exclusion criteria will be conducted by the study Principal Investigator (PI). Confirmation of eligibility with respect to pain scores will be ascertained by telephone review following screening.

Screen failures may be rescreened only where there is a short-term reason for ineligibility, such as an ongoing acute illness. A screening log will be kept on site to document details of patients invited to be screened for participation in the study. For patients who decline or are ineligible, this will document any reasons available for non-participation (where provided). The log will ensure potential participants are only approached once. The original signed consent form will be retained in the investigator site file, with a copy in the participant’s hospital medical notes and a copy provided to the participant. The participant will specifically consent to their general practitioner (GP) being informed of their participation in the study. The right to refuse to participate without giving reasons will be respected.

### Inclusion criteria

The inclusion criteria are:

- Chronic neuropathic pain following peripheral nerve injury, definite or probable (the grading of certainty for the presence of neuropathic pain includes definite neuropathic pain: all (1–4); probable neuropathic pain: 1 and 2, plus either 3 or 4 [1]:

1. Pain with a distinct neuroanatomically plausible distribution
2. A history suggestive of a relevant lesion or disease affecting the peripheral or central somatosensory system
3. Demonstration of the distinct neuroanatomically plausible distribution by at least one confirmatory test
4. Demonstration of the relevant lesion or disease by at least one confirmatory test.

- ≥ 12 months duration of pain. Post-traumatic nerve regeneration is usually complete at 12 months, so that inclusion of this group will reduce the likelihood of pain relief due to nerve regeneration [26].
- Adults aged 18 or older.
- Moderate to severe pain intensity: average 24-h pain intensity over 7 days at baseline of ≥ 5/10 but not dropping below 4 on any single day on an 11-point (0–10) NRS.
- Pain localised to the distribution of one to two peripheral nerves (to limit the time burden with respect to treatment application).
- Distribution of pain that will allow for the nerve to be stimulated proximally from the areas of pain. Invasive peripheral nerve stimulation (PNS) trial evidence suggests analgaesia is maximised when stimulation is used in this way [27].
- Medications that numb affected areas should be discontinued prior to the study: lidocaine patches 2 weeks prior, capsaicin treatments (at both low and high concentrations) 4 months prior to EN-PENS to allow nerve endings to grow back.
- Patients should have trialled first-line pharmacotherapy. First-line treatments include tricyclic antidepressants,serotonin-noradrenaline reuptake inhibitors, pregabalin or gabapentin [6].
- Moderate to severe brush stroke allodynia, defined as pain of ≥5/10 on an 11-point (0–10) NRS when a brush stroke is applied to the affected area (average of 3 strokes over affected area).
- Willingness to not commence any new medications/treatments for their neuropathic pain whilst involved in the trial.
- Women of childbearing potential may participate providing they are using adequate birth control methods for the duration of the trial. Included accepted methods of contraception are, e.g. barrier methods, intrauterine device (IUD), contraceptive implant, depot injection, oral contraception and abstinence (as part of lifestyle choice).

### Exclusion criteria

The exclusion criteria are:

- Absolute numbness. This suggests sufficient nerve damage that will render EN-PENS unlikely to work [28].
- Known EN-PENS contraindications:

- ○ Pregnancy; non-pregnancy will be confirmed by urine test at baseline and at treatment end (12 weeks)
- ○ Cardiac pacemakers.

- Other chronic pain or unstable medical conditions which in the opinion of the investigators would make the trial unsuitable for the patient.
- Unstable pain intensity or pain medications 6 weeks prior to the study that in the judgment of the PI would interfere with assessment of outcome.
- Persons participating in an interventional trial within the past 3 months.
- Persons participating in a non-interventional trial completed within the past 2 weeks.
- Diagnosed psychiatric or mental health disorder which could in the judgment of the PI interfere with successful study participation. This will be identified via patient self-report or from case notes.
- Inability to comply with the study protocol for the trial period of 3 months.
- Inability to complete outcome measures, e.g. impaired understanding.
- Inability to understand the information necessary to provide informed consent.
- Other implanted device for the same pain complaints such as spinal cord stimulator, dorsal root ganglion stimulator or deep brain stimulator.
- Phantom limb pain as the primary target for stimulation.

### Randomisation

Randomisation will be conducted by an independent randomisation service via an online system. Randomisation will use a 1:1 allocation by a computer-generated randomisation schedule (concealed). Blocking will be used to ensure a balance between the numbers in the two groups throughout recruitment. Varying block sizes will be used. Only site staff authorised to request randomisation will receive passwords for the randomisation system (PI and Trial Manager (TM)).

Prior to randomisation, pain diaries will be collected and scored to confirm study inclusion eligibility. Diary values override values ascertained over the phone, and patients will be excluded at this point if their diary values are not in keeping with the inclusion criteria.

### Blinding

Study trial nurses, who are blinded as to which is the active and which is the sham treatment, will be told the assigned allocation of the patient to either ‘flat electrode’ or ‘pen electrode’ so that they can assign the appropriate machine to the patient to commence patient training. Both the research nurses and patients will be able to see that there are different electrodes, and they will be informed that both interventions deliver electrical current to the nerve supplying the painful territory and that the purpose of this trial is to compare two types of electrical stimulation and find out how much they relieve pain.

### Stimulation interventions

In order to maintain blinding, it is important that control stimulation produces a perceivable but non-therapeutic electrical stimulation (patient focus group feedback). Control and active stimulation parameters were developed in discussion with our collaborator Professor Magerl (Universität Heidelberg, Center for Biomedicine and Medical Technology Mannheim, Germany) [13].

### Active and control interventions

The active and control machines will look identical. Both machines will deliver a low-frequency electrical current of 2 Hz. The machines will differ in terms of connecting electrode and parameters of amplitude and pulse width. One machine will use a ball-shaped electrode that will be placed onto the skin, whilst the other will use a flat square electrode placed onto the skin. The control machine will use a different amplitude and pulse width than the active machine. In the control devices the combination of the electrode shape and the different amplitude and pulse width will prevent electrical stimulation activating superficial nociceptive A-delta fibres inducing long-term depression (LTD) of synaptic strength [15, 28]. The display will appear to allow patients the same freedom to increase stimulation on both machines to 30 mA; however, the control devices’ delivered maximal output will be limited to 6 mA. As the sham control machine will deliver a small current, these control stimulation parameters were trialled on known EN-PENS responsive patients to confirm therapeutic non-efficacy.

In order to prevent unblinding, we have not provided further details, but the authors can be contacted to request further information.

### Stimulation training

#### Interventions training, visits 2–4

Patients will be taught to use the EN-PENS machine to which they have been allocated over one to three training sessions within a 1-week timeframe [19].

##### Identification of nerve to stimulate

In the first training session, an independent physiotherapist with experience using EN-PENS will determine the nerve to stimulate from the patient’s report of pain. This method is in keeping with both the technique used in the previous study [19] and literature regarding implantable PNS [9, 29]. The independent physiotherapist will have no further direct patient contact during the trial. A photograph will be taken within the first training session of the location of stimulation and will be used to support the research nurse and patient in subsequent training sessions and during the home loan period.

During training, subjects in both groups will be advised to increase the stimulation amplitude to just above a perceivable level. Patients will receive no more than 5 minutes of stimulation during training. The LTD effect would not be obtained within training even if the active machine was used, because effective LTD requires delivery of about 1200 pulses, typically obtained from 10 minutes of treatment [16]. No pain scores will be recorded pre- or post-treatment during training sessions.

#### Treatment phase (home loan), visit 3 or 4

Once the patient is considered to have achieved competence in using the EN-PENS device, he/she will commence the 3-month treatment period. Patients will be advised they need to stimulate for a minimum period of 10 minutes in order to achieve the LTD effect. They will use the device with fixed pulse frequency and pulse width, but will determine the treatment frequency and stimulation intensity for themselves. Patients will be advised that the stimulation amplitude should be mildly painful but not exceed an unpleasant level. They will also be told that this ‘mildly painful but not intolerable’ level will differ greatly between patients, with some patients reaching it at a low milliampere level, others at a higher milliampere level and yet others not at all. If this mildly painful level is not reached, maximal stimulation should nevertheless be maintained.

#### Optional treatment extension/swap, visits 4–5

On completion of the 3-month home loan, participants will be offered the choice of an optional treatment extension/treatment swap or termination of the study.

### Outcome measures

#### Primary outcome

The primary outcome is calculation of the average 24-h pain intensity recorded on an 11-point (0–10) numerical rating scale (NRS) [30], averaged over the last 7 days of the 3-month home loan period. The primary outcome will be assessed with daily completion during the 7-day screening period (between visit 1 and visit 2) and daily over the last 7 days of the home loan period.

Pain intensity will also be assessed with weekly completion of 7-day average pain intensities during the home loan period and the optional extension. This will be measured using pain diaries that also record treatment frequency in order to monitor study adherence.

Additionally any symptoms and adverse events (any undesirable symptom or experience associated with treatment) will be recorded.

#### Secondary outcomes

The secondary outcomes are:

- Brief Pain Inventory Interference Subscale (functional interference) score [31]
- Health-related quality of life questionnaire (EuroQol-5D-5 L) score [32]
- Completion of a modified Client Service Receipt Inventory (CSRI) (health care resource use) [33], supported by a regular telephone questionnaire [34].

Functional interference and health care resource use will be recorded at baseline and at 3 months. EuroQOL-5D-5 L score will be recorded at baseline and at 1, 2 and 3 months.

### Exploratory outcomes

Exploratory outcomes are:

- Hospital Anxiety and Depression Scale [35] (emotional function) score
- Pain Self-Efficacy Questionnaire [36] (perceived confidence to function despite pain) score
- Allodynia mapping (change in surface area of allodynia [37])
- Neuropathic Pain Symptom Inventory (NPSI) [38] (quality of pain) score
- A one-off specially designed sensory testing protocol (assessment of potential working mechanisms of EN-PENS), to be performed for approximately 40 patients who consent to testing
- Changes in medication consumption as they occur
- A case review by a named pain consultant specialising in spinal cord stimulation (potential medical suitability for invasive neuromodulation).

Exploratory measures will be recorded at baseline and at 3 months unless otherwise stated.

Outcomes noted on completion of the study only are:

- Patient-perceived global impression of change [39]
- Success of blinding [40].

### Definition of end of study

The end of the study will be the last participant’s final study contact at day 196 (+/–14 days, for those who participate in the open extension/treatment swap) or at day 112 (+/– 14 days, for those who elect not to enter the open extension/swap).

### Sample size

Within the previous study we observed a post-treatment NRS reduction of 6.4 to 3.6 (average difference 2.8) for the entire group (*n* = 20), and 6.6 to 3.1 (average difference 3.5) within the peripheral neuropathic pain group (*n* = 8) [19]. The mean observed effect for the treatment group would remain 3.5 only if the control group showed no change over time. There was no control group as part of this study; therefore, we estimated potential control group response based on best available literature. We were unable to identify any systematic reviews indicating baseline pain intensities and placebo responses specifically in RCTs for post-injury neuropathic pain. Therefore, data from a systematic review and meta-analysis on a different post-traumatic neuropathic pain condition, complex regional pain syndrome, was used [41]. In this systematic review the pooled mean at baseline for pain (17 studies assessed in the non-active arms) was 6.4. The systematic review illustrated no significant evidence of placebo response at time points comparable to our proposed trial. Given the observed effect size from our published audit, the proposed group difference of 1.5 NRS provides a conservative estimate in relation to between-group effect size differences.

It is conservatively estimated that there is a correlation of 0.5 between the baseline and outcome pain scores; in our audit the correlation was 0.64. With a 5% significance level and 90% power, 26 participants per group are required. Allowing for a 30% dropout, based on results from a prospective patient audit, a sample size of 38 participants per group is required, 76 in total.

### Data handling

Stored data will be coded and anonymised information and will be stored securely: all electronic data will be password protected. Hard copy data will be stored in a locked filing cabinet in a secure office for 5 years.

### Statistical analysis

Data analysis will be according to the intention-to-treat principle. The primary analysis will be of observed data only, with missing values omitted from the analysis. Therefore, patients who do not contribute any outcome data will not be analysed. The primary outcome will be analysed using analysis of covariance (ANCOVA). Baseline pain scores will be considered as a covariate in the analysis. The mean difference in outcome between the two groups will be reported with 95% confidence intervals. As a secondary analysis of the primary outcome, the pain scores will also be considered an ordinal outcome (as opposed to continuous for the primary analysis).

A sensitivity analysis will be performed using multiple imputation to address missing values. The extent of missing values will be investigated, as will the nature of variables with missing data. For the sensitivity analysis, multiple imputation will be performed based on either the multivariate normal data distribution [42] or using chained equations [43]. Ordinal logistic regression will be used to compare between groups as an additional sensitivity analysis [44].

Secondary and exploratory continuous outcomes will be analysed using ANCOVA as with the primary outcome [45]. For binary outcomes the number and percentage of patients experiencing the event for each treatment arm will be presented. The relative risk and 95% confidence interval will be presented, and the chi-squared test will be used to determine statistical significance [46]. Adverse events will be analysed using descriptive statistics only, as there is unlikely to be sufficient power to formally compare occurrence.

The occurrence and reasons for missing data will be examined via multiple imputation methods to analyse factors such as self-efficacy in relation to adherence. Additionally, a post hoc analysis will be used to assess whether there is a correlation between efficacy and symptom profiling using the specified Neuropathic Pain Symptom Inventory.

### Health economic analysis

There are no economic analyses of EN-PENS relevant to a UK context. A de novo prospective economic evaluation is therefore warranted, given the cost differential between the interventions being evaluated and the potential for clinical benefits (should the null hypothesis be disproved). The health economic analysis will adopt the perspective of the National Health Service (NHS) and Personal Social Services. Costs will include those of treatment, procedures and investigations, contact with primary and secondary care services and personal social services. Resource use will be obtained from patients’ self-reporting of resource use, captured by telephone questionnaire administration [47] and retrospective case report form. Unit cost data will be obtained from standard sources [33, 48, 49]. The primary economic outcomes will be the incremental cost per quality-adjusted life year (QALY) gained, estimated by administering the EQ-5D-5 L. The number of QALYs experienced by each patient will be calculated as the area under the curve, using the trapezoidal rule, applying the UK tariffs. Total costs will be combined with QALYs to calculate the incremental cost-utility ratio, which will be compared with the £20,000 to £30,000 per QALY threshold of cost-effectiveness. A range of one-way sensitivity analyses will be conducted to assess the robustness of the analysis, and multivariate sensitivity analyses will be applied where interaction effects are suspected. The joint uncertainty in costs and benefits will be considered through the application of bootstrapping and the estimation of cost-effectiveness acceptability curves [50].

### Withdrawal of participants

The study treatment must be discontinued and patients withdrawn if:

- Participants decide they no longer wish to continue (see also below)
- Recommended by the Investigator or another clinician (e.g. due to intercurrent illness during course of study or increased pain following repeated stimulation)
- The trial is terminated if deemed appropriate by the study staff in consultation with the study’s PI and pain consultant.

Where appropriate, patients who wish to discontinue their study intervention or for whom discontinuation is advised will be asked whether they would be willing to continue in the study by providing weekly diaries.

Patients are also withdrawn if:

- They are randomised but never receive any treatment (i.e. the first training session is never started — this is also termed ‘non-compliance’)
- They fail to complete weekly pain diary reports on three consecutive occasions or 4 nonconsecutive weeks that include the last month of treatment (this is also termed ‘missing data’).

All data from patients randomised to treatment will be included in the intention-to-treat analysis (see ‘Statistical analysis’).

Participants have the right to withdraw from the study at any time without providing a reason. The Investigator also has the right to withdraw a participant from the study if they consider that it is in the best interests of the participant (adverse events). Should a participant decide to withdraw from the study, he/she will be asked to volunteer a reason for withdrawal.

Subjects who withdraw from treatment early will be encouraged to return to the study site to have follow-up outcomes (week 13), providing that consent is not withdrawn. Participants who withdraw early will be expected to return all study equipment within 2 weeks of ending the study.

## Trial organisation and monitoring

The EN-PENS protocol has been extensively reviewed by clinicians, statisticians and patient groups. The trial has been registered with an International Standard Randomised Controlled Trial Number (ISRCTN), registration number ISRCTN53432663. All aspects of trial administration will be conducted by the Sponsor site Neurosciences Research Unit and finance departments.

An external project monitor will also be appointed as part of data quality assurance. The Neurosciences Research Unit trials manager will supervise Neurosciences Research Unit staff.

Day-to-day trial management by the TM and PI will include establishing and carrying out the trial in accordance with international, national and local laws and regulations and good clinical practice (GCP). PI mentorship and support will be provided by pain consultants AG and TJ Nurmikko.

Quarterly trial management meetings will review the day-to-day running and management of the trial. Members will include the PI, TM, Data Manager, health economists (DH and EH), trial statistician, pain consultant AG, and consumer representative Wendy Hall.

The trial management group will report to a trial steering group who will provide overall supervision of the trial and ensure milestones are met and the trial adheres to Medical Research Council (MRC) Guidelines for Good Clinical Practice in Clinical Trials.

The trial steering group will comprise the PI, the TM and five to six persons independent of the trial running. It will also include a consumer representative.

Annual reports will be presented by the PI to the centres, research governance committee and pain services management group and National Institute for Health Research (NIHR) funders.

The study may also be subject to audit or inspection by the University of Liverpool or the Walton NHS Trust under their remit as Co-sponsors, or by the Medical Health Research Authority (MHRA) or other regulatory bodies to ensure adherence to GCP and regulatory requirements.

## Direct access to source data and documents

The investigators agree to provide full access to all source data, study data and materials to the trust research governance department, ethics committee, regulatory authority and Trial Manager for purposes of monitoring, audit or inspection.

## Expected adverse reactions

Skin irritation may be expected in persons who demonstrate sensitivity to aqueous gel or electrode pads. These adverse events are expected in the sense that they are possible known side effects of the study intervention. All reported instances of both serious and non-serious adverse events will be reported in this study.

### Recording and reporting non-serious adverse events and serious adverse events/reactions (including suspected unexpected serious adverse reactions)

All adverse events and all serious adverse events (SAEs) should be recorded. All non-serious adverse events will be recorded on the study case report forms. Relation of any adverse events to treatment should be assessed by the trials pain consultant. All SAEs, serious adverse reactions (SARs) and suspected unexpected serious adverse reactions (SUSARs) shall be recorded and reported on the SAE form to the trial medic and PI within 24 hours of learning of its occurrence.

Relationship to the treatment will be assessed by the trials pain consultant. As this is a blinded trial involving a control and active treatment, seriousness, causality and expectedness should be evaluated as though the patient were on the active treatment. The Sponsor will report SUSARs and other SARs to the regulatory authority (MHRA) in accordance with the reporting timelines.

The PI will report to the North West Coast - Preston ethics committee.

The PI is unblinded throughout the study; therefore, reporting of incidents will not affect the integrity of the trial blinding.

### Publication policy

Results from the study will be submitted for publication by the investigators only, in international medical journals. Results of the study will also be reported to the Sponsor and Funder in the required format.

Participants will be provided with a summary of the results once the primary paper has been accepted for publication.

## Discussion

This protocol will allow us to determine if external non-invasive peripheral nerve stimulation is an effective treatment for neuropathic pain following peripheral nerve injury. The aim is to have 76 patients recruited by 30 August 2018 with the write-up completed by 30 August 2019.

## Trial status

The current status is open, the closure date is 30 August 2018 and the global sample size is 76.

## Abbreviations

CI: Confidence interval
CRPS: Complex regional pain syndrome
DBS: Deep brain stimulation
DRG: dorsal root ganglion (stimulation)
EN-PENS: External non-invasive peripheral nerve stimulation
GCP: Good clinical practice
GMC: General medical council
Hz: Hertz
LTD: Long-term depression
mA: Milliampere
MHRA: Medical Health Research Authority
ms: millisecond
NHS: National Health Service
NIHR: National Institute for Health Research
NPSI: Neuropathic Pain Symptom Inventory
NRES: National Research Ethics Service
PENS: Percutaneous electrical peripheral nerve stimulation
PI: Principal Investigator
PPNI: Pain post peripheral nerve injury
QOL: Quality of life
RCT: Randomised controlled trial
RfPB: Research for Patient Benefit
SAE: Serious adverse event
SAR: Serious adverse reaction
SCS: Spinal cord stimulation
SUSAR: Suspected unexpected serious adverse reaction
TENS: Transcutaneous electrical nerve stimulation
TM: Trial Manager
UK: United Kingdom
